# Duck Enteritis Virus Protein Kinase US3 Inhibits DNA Sensing Signaling by Phosphorylating Interferon Regulatory Factor 7

**DOI:** 10.1128/spectrum.02299-22

**Published:** 2022-10-26

**Authors:** Rui Liu, Li Gao, Fuchun Yang, Xiaohan Li, Changjun Liu, Xiaole Qi, Hongyu Cui, Yanping Zhang, Suyan Wang, Xiaomei Wang, Yulong Gao, Kai Li

**Affiliations:** a Division of Avian Immunosuppressive Diseases, State Key Laboratory of Veterinary Biotechnology, Harbin Veterinary Research Institute, Chinese Academy of Agricultural Sciences, Harbin, China; b Jiangsu Co-innovation Center for Prevention and Control of Important Animal Infectious Disease and Zoonoses, Yangzhou University, Yangzhou, China; Wayne State University

**Keywords:** duck enteritis virus, DNA sensing, interferon regulatory factor 7, US3

## Abstract

The cytosolic DNA sensing pathway mediates innate immune defense against infection by many DNA viruses; however, viruses have evolved multiple strategies to evade the host immune response. Duck enteritis virus (DEV) causes an acute and contagious disease with high mortality in waterfowl. The mechanisms employed by DEV to block the DNA sensing pathway are not well understood. Here, we sought to investigate the role of DEV US3, a serine/threonine protein kinase, in the inhibition of DNA sensing. We found that ectopic expression of DEV US3 significantly inhibited the production of IFN-β and expression of interferon-stimulated genes induced by interferon-stimulatory DNA and poly(dA-dT). US3 also inhibited viral DNA-triggered IFN-β activation and promoted DEV replication in duck embryo fibroblasts, while knockdown of US3 during DEV infection enhances the IFN-β response and suppresses viral replication. US3 inhibited the DNA-sensing signaling pathway by targeting interferon regulatory factor 7 (IRF7), and the kinase activity of US3 was indispensable for its inhibitory function. Furthermore, we found that US3 interacts with the activation domain of IRF7, phosphorylating IRF7, blocking its dimerization and nuclear translocation, and finally leading to the inhibition of IFN-β production. These findings expand our knowledge on DNA sensing in ducks and reveal a novel mechanism whereby DEV evades host antiviral immunity.

**IMPORTANCE** Duck enteritis virus (DEV) is a duck alphaherpesvirus that causes an acute and contagious disease with high mortality, resulting in substantial economic losses in the commercial waterfowl industry. The evasion of DNA-sensing pathway-mediated antiviral innate immunity is essential for the persistent infection and replication for many DNA viruses. However, the strategies used by DEV to block the DNA-sensing pathway are not well understood. In this study, DEV US3 protein kinase was demonstrated to inhibit the DNA-sensing signaling via binding to the activation domain of interferon regulatory factor 7 (IRF7), which induced the hyperphosphorylation of IRF7 and abolished IRF7 dimerization and nuclear translocation. Our findings provide insights into how duck herpesviral kinase counteracts host antiviral innate immunity to ensure viral replication and spread.

## INTRODUCTION

Duck enteritis virus (DEV), also known as Anatid herpesvirus type 1, causes an acute and contagious disease with high morbidity and mortality in domestic waterfowl, resulting in severe economic losses to the duck industry (1). DEV is a member of the family *Herpesviridae* and subfamily *Alphaherpesvirinae*. This virus has a worldwide distribution, and migratory waterfowl play a crucial role in its transmission within and between continents. Domestic and wild ducks, geese, and swans of all ages are considered susceptible to DEV infection. DEV is considered a pantropic virus that replicates quickly in many cell types and tissues, leading to pathological lesions in many different organs (2). It replicates primarily in the mucosa of the digestive tract and then spreads to the bursa of Fabricius, thymus, spleen, and liver. This virus exhibits latent infection in trigeminal ganglia after the establishment of primary infection in ducks; thereafter, viral reactivation results in disease outbreak (3). The DEV genome is approximately 158 kb and contains 78 open reading frames predicted to encode potential functional proteins. Despite many advances in the understanding of DEV pathogenesis, little is known about the innate immune responses and the virus-host interaction during DEV infection in ducks.

The type I interferon (IFN-I) signaling pathway acts as the first line of host defense by inducing a wide range of antiviral effectors to eliminate invading pathogens (4, 5). The activation of the IFN-I pathway depends on the recognition of viral constituents by host pattern recognition receptors (PRRs), which ultimately induces the expression of multiple IFN-stimulated genes (ISGs) and instigates innate antiviral responses. The best-known PRRs that recognize viral infection are the Toll-like receptors (TLRs), RIG-I-like receptors, and cytosolic DNA receptors (5–7). The recognition of viral DNA by DNA sensors is a central host cellular defense against DNA virus infection. Among the identified DNA sensors, cyclic GMP-AMP synthase (cGAS) is the predominant cytosolic DNA sensor recognizing a variety of DNA ligands in different cell types (8). Upon binding to viral DNA, cGAS synthesizes the second messenger cyclic GMP-AMP (cGAMP) to activate stimulator of interferon genes (STING). Active STING then recruits TANK-binding kinase 1 (TBK1) to phosphorylate and activate interferon regulatory factor 3 (IRF3). Phosphorylated IRF3 dimerizes and then translocates into the nucleus, ultimately leading to the production of IFN-I and several inflammatory cytokines (9, 10). Recently, the cGAS-STING DNA sensing pathway was reported to play an important role in IFN-I responses against herpesviruses, including herpes simplex virus 1 (HSV-1), Kaposi sarcoma herpesvirus, and Marek’s disease virus (11–13). Evasion of the host antiviral innate immunity is essential for herpesviruses to establish persistent infection and replication in the host (14). Moreover, several viral proteins that inhibit IFN-I production through modulation of the DNA sensing signaling pathway have been identified (15–17).

Birds, including chickens, ducks, and geese, are natural reservoirs for many kinds of viruses. IFN-I has been characterized and assessed for its antiviral activities in various avian species. Duck IFN-I was first detected in duck embryo fibroblasts (DEFs) after infection with reovirus serotype 3 (18). Avian PRRs differ from their mammalian counterparts (18). To date, four duck TLRs have been identified, including TLR2, TLR3, TLR4, and TLR7. Comparatively, chicken, duck, and goose cGAS exhibit shortened N termini with 22.4%, 17.4%, and 7.7% similarity to human cGAS, respectively (19, 20). As in mammals, the cGAS-STING axis plays a critical role in restricting DNA virus infection in chicken and duck cells (21, 22). Recently, the DEV VP16 protein was shown to inhibit DNA sensing by interacting with duck IRF7 (22). However, it remains unclear whether other DEV viral proteins are involved in the antagonization of the DNA sensing pathway.

DEV encodes US3, a serine/threonine protein kinase that is conserved in the subfamily *Alphaherpesvirinae*. HSV-1 US3 is essential in multiple processes during viral infection, including viral genome replication, nuclear egress, virion maturation, apoptosis inhibition, cell-to-cell spread, and cytoskeletal rearrangements (23–27). HSV-1 US3 also participates in viral immune evasion by inhibiting IFN-I production and downregulating major histocompatibility complex class I surface expression (28–30). However, the biological functions of DEV US3 and its substrates have not been studied in detail. In this study, we aimed to identify the cellular substrates of DEV US3 in the DNA sensing pathway and to investigate the role of DEV US3 in the evasion of host innate immunity. Our results show that DEV US3 interacts with and hyperphosphorylates IRF7, which suppresses the dimerization and nuclear translocation of IRF7, thereby leading to a blockade of IFN-I production. Overall, our findings reveal a novel mechanism whereby DEV evades host antiviral immunity.

## RESULTS

### DEV US3 inhibits IFN-β production induced by interferon-stimulatory DNA.

**FIG 1:**
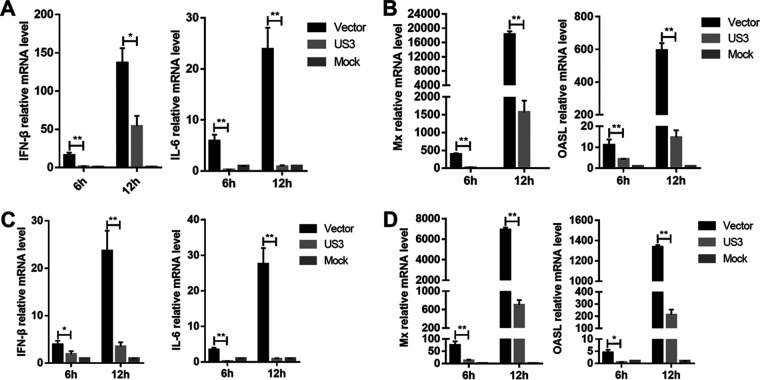
DEV US3 inhibits IFN-β production induced by ISD and poly(dA-dT). (A and B) DEFs were transfected with US3 expression plasmid or empty vector for 24 h and then transfected with ISD (1 μg/mL); the mRNA levels of *IFN-β*, *IL-6*, *Mx*, and *OASL* were measured by qPCR 6 h and 12 h later. (C and D) DEFs were transfected with US3 expression plasmid or empty vector for 24 h and then transfected with poly(dA-dT) (1 μg/mL); the mRNA levels of *IFN-β*, *IL-6*, *Mx*, and *OASL* were measured by qPCR 6 h and 12 h later. The relative amounts of *IFN-β*, *IL-6*, *Mx*, and *OASL* mRNA were normalized to *β-actin* mRNA levels in each sample, and the fold differences between the treated samples and the mock controls were calculated. All controls and treated groups were tested in triplicate. *, *P* < 0.05; **, *P* < 0.01.

To verify the role of DEV US3 in the regulation of IFN-β production induced by cytosolic DNA, DEFs were transfected with a US3 expression plasmid or empty vector (EV); after 24 h, they were transfected with interferon-stimulatory DNA (ISD) fragments, which induce IFN-β expression in various cells. As shown in Fig. 1A, transfection with ISD induced high levels of IFN-β and IL-6 mRNAs in DEFs; however, ectopic expression of US3 markedly inhibited ISD-triggered IFN-β and IL-6 mRNA expression. Furthermore, ectopic expression of US3 also significantly inhibited ISD-induced expression of ISGs, including myxovirus resistance protein (Mx) and interferon-induced oligoadenylate synthetase-like (OASL) (Fig. 1B). Poly(dA-dT) is a synthetic analog of B-DNA which is recognized by several DNA sensors. As shown in Fig. 1C, the IFN-β and interleukin 6 (IL-6) mRNA levels were significantly increased in EV-transfected cells in response to poly(dA-dT) stimuli, and this effect was attenuated in US3-expressing DEFs. In addition, the production of Mx and OASL induced by poly(dA-dT) was also reduced by the ectopic expression of US3 in DEFs (Fig. 1D). These results suggested that US3 inhibits the cytosolic DNA-induced production of IFN-β and ISGs in DEFs.

### DEV US3 suppresses viral DNA-triggered IFN-β production.

**FIG 2:**
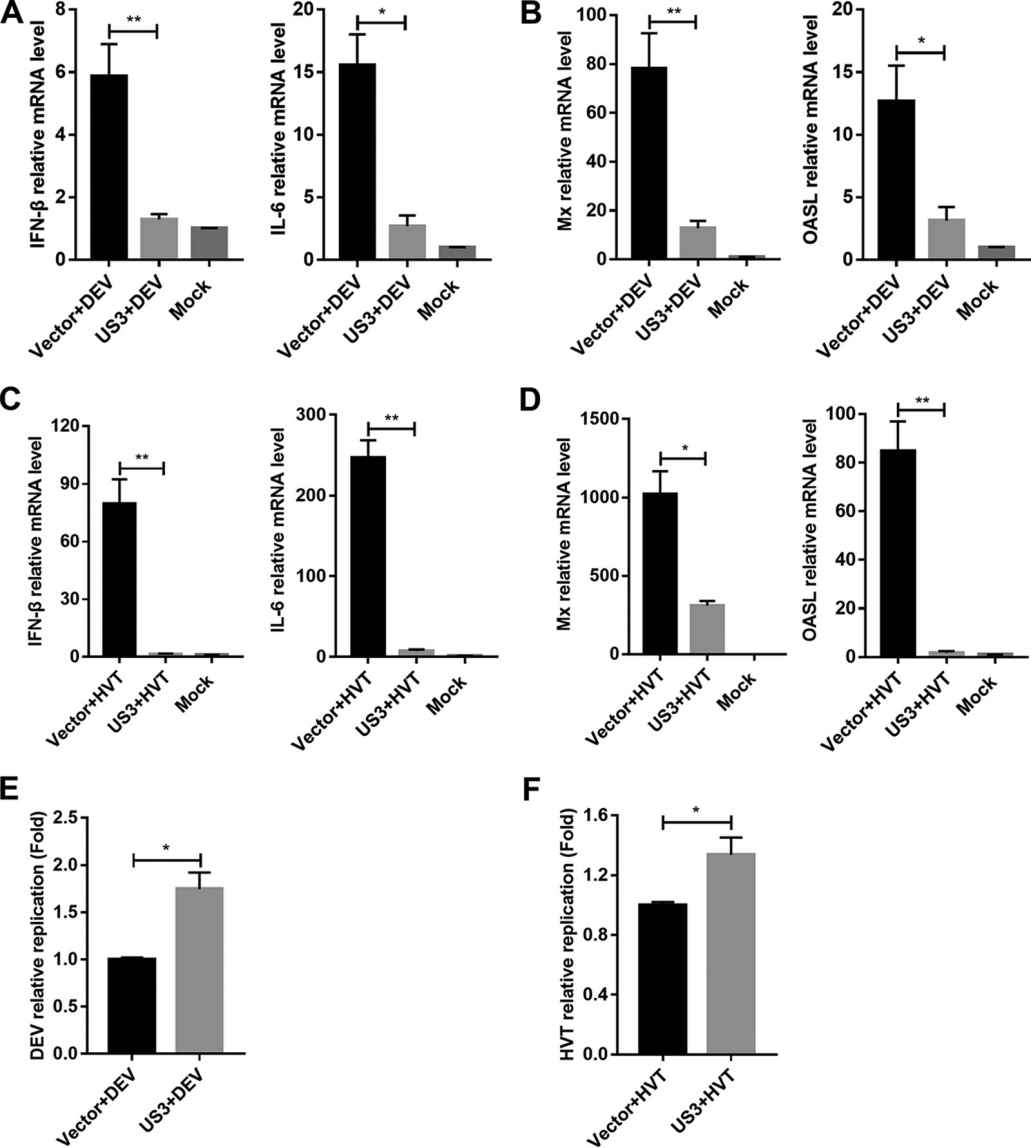
DEV US3 suppresses viral-DNA-triggered IFN-β production. (A and B) DEFs were transfected with US3 expression plasmid or empty vector for 24 h and then left uninfected or infected with DEV (multiplicity of infection [MOI] = 0.1). The mRNA levels of *IFN-β*, *IL-6*, *Mx*, and *OASL* in these cells were measured by qPCR 12 h postinfection. (C and D) DEFs were transfected with US3 expression plasmid or empty vector for 24 h and then left uninfected or infected with HVT (MOI = 0.1). The mRNA levels of *IFN-β*, *IL-6*, *Mx*, and *OASL* in these cells were measured by qPCR 12 h postinfection. (E) DEFs were transfected with US3 plasmid or empty vector for 24 h and then infected with DEV (MOI = 0.01). The viral titer was tested by qPCR 48 h postinfection. (F) DEFs were transfected with US3 plasmid or empty vector for 24 h and then infected with HVT (MOI = 0.01). The viral titer was tested by qPCR 48 h postinfection. The relative amounts of *IFN-β*, *IL-6*, *Mx*, and *OASL* mRNA were normalized to *β-actin* mRNA levels in each sample, and the relative amounts of DEV and HVT genomic DNA were normalized to *GAPDH* levels in each sample. The fold differences between the treated samples and the mock controls were calculated. All controls and treated groups were tested in triplicate. *, *P* < 0.05; **, *P* < 0.01.

Next, we tested the effects of US3 ectopic expression on the production of IFN-β triggered by DNA virus infection. The US3-expressing cells were infected with DEV, and the IFN-β and IL-6 mRNA levels were evaluated using quantitative real-time PCR (qPCR). The results showed that, compared with the EV-transfected control cells, US3-expressing cells had a reduced IFN-β response against DEV (Fig. 2A). The production of IL-6, Mx, and OASL was also reduced by US3 overexpression during DEV infection in DEFs (Fig. 2A and B). We also infected US3-expressing DEFs with herpesvirus of turkey (HVT) and found that US3 expression led to a diminished production of IFN-β and IL-6 in HVT-infected cells, compared to that observed in control cells (Fig. 2C). US3 overexpression also inhibited the expression of Mx and OASL induced by HVT infection (Fig. 2D). Consistently, both DEV and HVT exhibited higher genome copy numbers in US3-expressing cells (Fig. 2E and F). Taken together, these results indicated that DEV US3 inhibits viral DNA-triggered IFN-β activation and promotes viral replication.

### DEV US3 inhibits cGAS-STING-mediated IFN-β activation by targeting IRF7.

**FIG 3:**
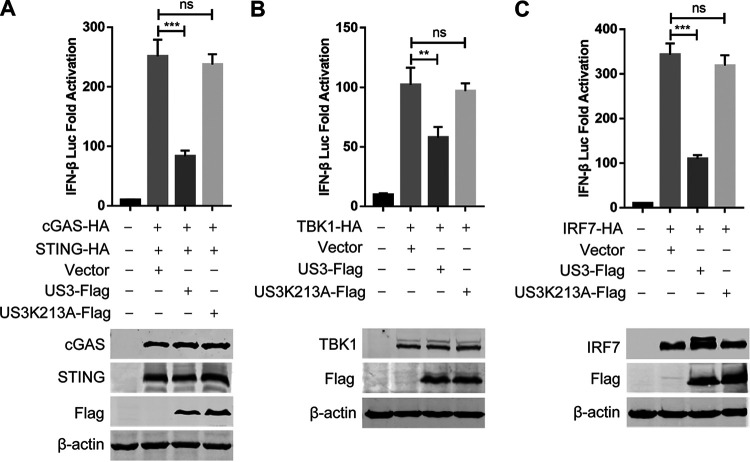
DEV US3 inhibits cGAS-STING-mediated IFN-β activation by targeting IRF7. (A) The cGAS and STING expression plasmids were cotransfected with the IFN-β-luc reporter, as well as the plasmid expressing wild-type US3 or US3 mutant US3K213A or empty vector, into DEFs; 24 h later, cells were subjected to the dual-luciferase reporter assay. (B and C) IFN-β-luc reporter was transfected with plasmids expressing TBK1 (B) or IRF7 (C) together with the plasmid expressing wild-type US3 or US3K213A mutant or empty vector into DEFs. Dual-luciferase reporter assays were performed 24 h posttransfection, and protein expression was determined by Western blotting. Data are means and SDs from three independent experiments. ns, no significant difference; **, *P* < 0.01; ***, *P* < 0.001.

To determine whether US3 can inhibit cGAS-STING-mediated IFN-β production, the US3 expression plasmid was transfected into DEFs along with the cGAS and STING expression plasmids and the duck IFN-β promoter reporter. As shown in Fig. 3A, the ectopic expression of US3 significantly inhibited cGAS-STING-mediated activation of the IFN-β promoter. As ducks are IRF3 deficient, the transcription of IFN-β in duck cells is dependent on the binding of IRF7 to distinct regulatory domains in the promoter. To clarify the components in the cGAS-STING pathway targeted by US3, DEFs were cotransfected with the US3 expression plasmid, IFN-β Luc reporter plasmid, and expression plasmids encoding IRF7 signaling pathway components, including TBK1 and IRF7. Both TBK1 and IRF7 elicited IFN-β Luc reporter activity; however, US3 inhibited the IFN-β Luc activity triggered by all constructs (Fig. 3B and C). These findings indicated that DEV US3 probably suppresses the cGAS-STING DNA sensing pathway by targeting IRF7.

To determine whether the kinase activity of DEV US3 is required for the inhibition of DNA sensing signaling, we generated the kinase-dead US3 mutant US3K213A by mutating the lysine at position 213 to alanine, as described for the kinase-dead US3 mutant of HSV-1 (28) and Marek’s disease virus (31). The results showed that ectopic expression of the US3K213A mutant failed to inhibit the IFN-β promoter activation induced by cGAS-STING, TBK1, and IRF7 (Fig. 2A to C). These results suggested that the kinase activity of US3 is essential for inhibiting DNA sensing signaling.

### DEV US3 phosphorylates IRF7 and blocks IRF7 nuclear translocation.

**FIG 4:**
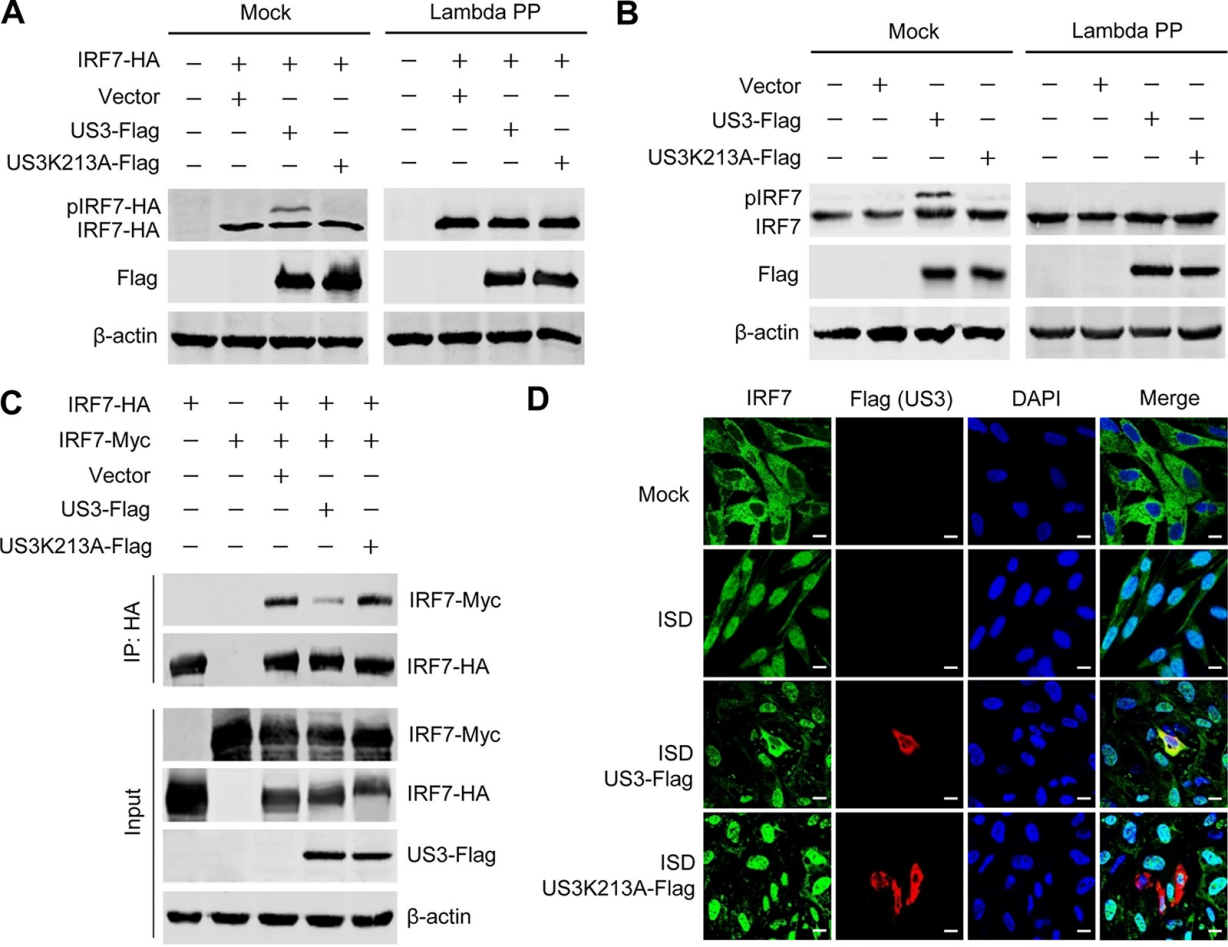
DEV US3 phosphorylates IRF7 and blocks IRF7 dimerization and nuclear translocation. (A) IRF7-HA was cotransfected with empty vector or US3- or US3K213A-expressing plasmid into DEFs. The cells were lysed 36 h later and left untreated or treated with lambda PP prior to SDS-PAGE and Western blotting. (B) DEFs were transfected with empty vector or US3- or US3K213A-expressing plasmid. The cells were lysed 36 h later, and Western blotting was performed with anti-IRF7 antibodies to detect IRF7 phosphorylation. (C) DEFs were transfected with the indicated plasmids for 36 h before coimmunoprecipitation and immunoblot analysis with the indicated antibodies. (D) DEFs were transfected with US3 or US3K213A plasmid; 24 h later, cells were either left untreated or transfected with ISD for 12 h before confocal microscopy. Bars, 10 μm.

The above data demonstrated that US3 targets IRF7 and that the kinase activity is required for US3-mediated inhibition of the DNA sensing pathway. Therefore, we investigated whether US3 phosphorylates IRF7. We transfected DEFs with the IRF7-hemagglutinin (HA) expression plasmid and the wild-type or kinase-dead mutant of US3 expression plasmids, and then the transfected cells were subjected to Western blot analysis. The results indicated that the expression of wild-type US3, but not the US3K213A mutant, induced an additional slowly migrating form of IRF7 (Fig. 4A). To determine whether the slowly migrating form of IRF7 was the result of US3-induced phosphorylation, we performed a dephosphorylation assay using lambda protein phosphatase (lambda PP). The slowly migrating form of IRF7 was eliminated following lambda PP treatment (Fig. 4A), indicating that the DEV US3-induced modification of IRF7 is due to phosphorylation. Moreover, endogenous duck IRF7 was also phosphorylated by the wild-type US3, but not the US3K213A mutant (Fig. 4B).

Dimerization and nuclear accumulation are hallmarks of IRF7 activation. Since US3 phosphorylated IRF7, we next examined whether the dimerization and nuclear translocation of IRF7 were affected by US3. As shown in the coimmunoprecipitation assay, IRF7 dimerization was markedly inhibited in the presence of US3, while the kinase-dead mutant US3K213A did not significantly affect IRF7 dimerization (Fig. 4C). Next, we transfected DEFs with the expression plasmid expressing US3 or US3K213A and monitored the effects of US3 on the ISD-induced nuclear accumulation of endogenous IRF7. The results showed that stimulation with ISD increased the IRF7 levels in the nuclei. However, ectopic expression of US3, but not the US3K213A mutant, prevented ISD-stimulated nuclear trafficking of IRF7 (Fig. 4D). These results indicated that DEV US3 phosphorylates IRF7 and blocks IRF7 dimerization and nuclear translocation.

### DEV US3 interacts with the activation domain of IRF7.

**FIG 5:**
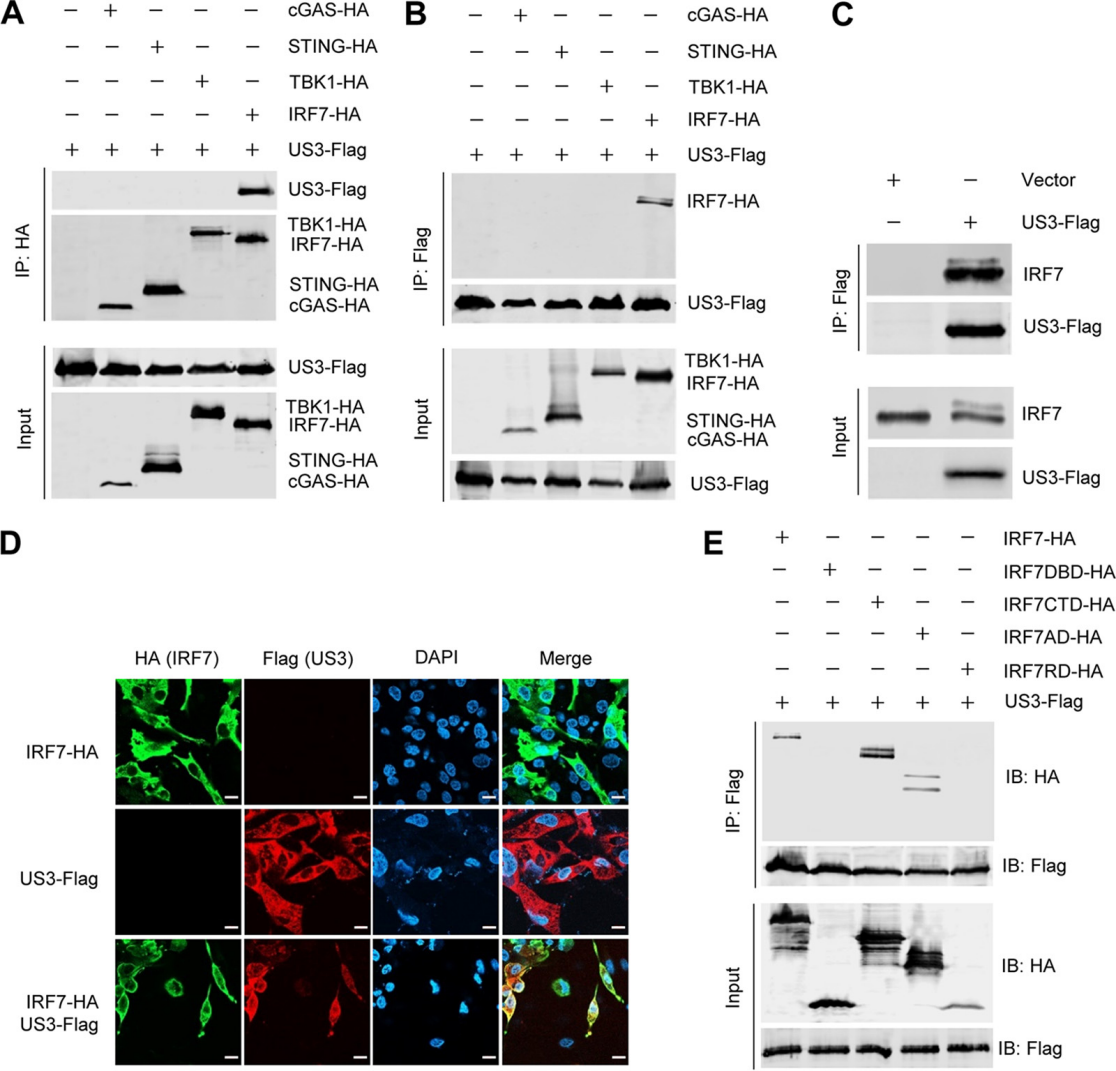
DEV US3 interacts with the activation domain of IRF7. (A and B) DEFs were transfected with the indicated plasmids for 36 h before coimmunoprecipitation and immunoblot analyses with the indicated antibodies. (C) DEFs were transfected with a US3-Flag expression plasmid or an empty vector; 36 h posttransfection, a coimmunoprecipitation assay was performed with anti-Flag antibody. (D) DEF cells were transfected with US3-Flag and/or IRF7-HA expression plasmids for 24 h and then fixed and processed for dual labeling. Cell nuclei were counterstained with DAPI (blue). US3 (red) and IRF7 (green) proteins were visualized by immunostaining with anti-Flag and anti-HA antibodies, respectively. Bars, 10 μm. (E) A US3-Flag expression plasmid was cotransfected with IRF7 (aa 1 to 512) or a series of truncation mutants of IRF7, including IRF7DBD (aa 1 to 141), IRF7CTD (aa 142 to 512), IRF7AD (aa 142 to 320), and IRF7IRD (aa 321 to 512), into HEK293T cells; 36 h posttransfection, the cell lysates were immunoprecipitated with anti-Flag antibodies and analyzed by Western blotting.

To elucidate the molecular mechanisms whereby US3 suppresses the DNA sensing pathway, coimmunoprecipitation assays were performed to investigate the interaction between US3 and the signaling pathway components. DEFs were transfected with US3-Flag along with cGAS-HA, STING-HA, TBK1-HA, or IRF7-HA, and a coimmunoprecipitation assay was performed with anti-HA and anti-Flag antibodies. We found that US3 was immunoprecipitated by IRF7 but not by cGAS, STING, or TBK1. Reciprocally, IRF7 was immunoprecipitated by US3 (Fig. 5A and B). Furthermore, the endogenous IRF7 was also immunoprecipitated by US3 (Fig. 5C). The interaction of US3 with IRF7 was additionally confirmed by their subcellular colocalization (Fig. 5D).

To identify the domains of IRF7 that are involved in its interaction with US3, we constructed a series of truncation mutants of duck IRF7, including IRF7DBD (amino-terminal DNA-binding domain, amino acids [aa] 1 to 141), IRF7CTD (carboxyl-terminal domain, aa 142 to 512), IRF7AD (constitutive and virus activation domain, aa 142 to 320), and IRF7IRD (inhibitory and signal response domain; aa 321 to 512). As shown in Fig. 5E, IRF7, IRF7CTD, and IRF7AD coimmunoprecipitated with US3, whereas IRF7DBD and IRF7IRD did not, suggesting that the constitutive and virus activation domain of IRF7 (aa 142 to 320) is essential for its association with US3. These results suggested that US3 interacts with and phosphorylates the activation domain of IRF7, which blocks the dimerization and nuclear translocation of IRF7, leading to the inhibition of DNA sensing signaling.

### US3 deficiency enhances IFN-β production and suppresses DEV replication.

**FIG 6:**
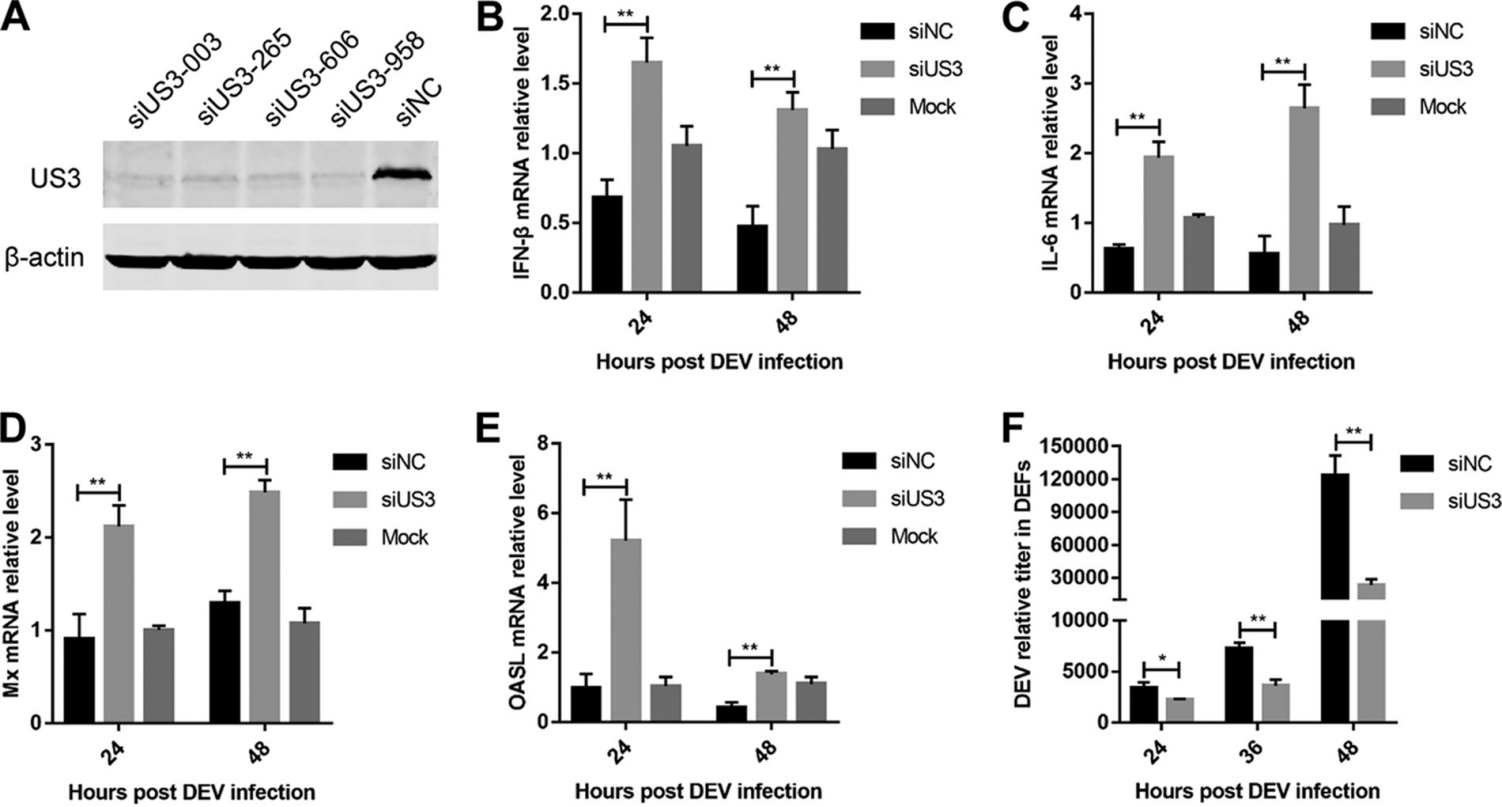
Knockdown of US3 enhances IFN-β production and attenuates DEV replication. (A) Western blot analysis of the DEFs transfected with US3-specific short hairpin RNA (shRNA) (siUS3-003, siUS3-265, siUS3-606, and siUS3-958) or a negative-control shRNA (siNC) after 36 h of DEV infection. (B to E) DEFs transfected with siNC or siUS3-003 were infected with DEV (MOI = 0.01). The mRNA levels of *IFN-β* (B), *IL-6* (C), *Mx* (D), and *OASL* (E) were measured by real-time qPCR. (F) DEFs transfected with siNC or siUS3-003 were infected with DEV (MOI = 0.01). The DEV viral titers were tested by real-time qPCR. The relative amount of *IFN-β*, *IL-6*, *Mx*, and *OASL* mRNA was normalized to *β-actin* mRNA levels in each sample, and the relative amount of DEV genomic DNA was normalized to *GAPDH* levels in each sample. The fold difference relative to the mock controls at each time point was determined. All controls and treated groups were tested in triplicate. *, *P* < 0.05; **, *P* < 0.01.

To investigate the roles of endogenous US3 in the antiviral response to DEV, we transfected DEFs with US3-specific small interfering RNA (siRNA). The Western blot results showed that all the tested US3-specific siRNAs effectively downregulated US3 expression in DEV-infected DEFs (Fig. 6A). DEFs were transfected with US3-siRNA-003 prior to infection, and then the DEV replication was compared with that in cells that had been transfected with a negative-control siRNA. The results showed that US3 knockdown promoted the induction of IFN-β and IL-6 in response to DEV infection at 24 and 48 h postinfection (hpi) (Fig. 6B and C). Moreover, transcription of the duck ISGs Mx and OASL induced by DEV infection was markedly increased in US3 knockdown cells compared with that in cells transduced with control siRNA (Fig. 6D and E). In addition, DEV replication was significantly suppressed in the US3 knockdown DEFs (Fig. 6F). These data suggest that knockdown of US3 during DEV infection increases IFN-β production and suppresses viral replication.

## DISCUSSION

During herpesvirus infection, viral DNA released into the cytoplasm triggers the activation of the cGAS-STING DNA sensing pathway, which results in the production of IFN-I and inflammatory chemokines to suppress viral replication (6, 7). Given the key role of the cGAS-STING pathway in the regulation of the host antiviral immune response, many viruses have evolved various mechanisms that target this signaling pathway to subvert the host innate immunity (32, 33). Several viral proteins are known to inhibit IFN-I production by modulating the cGAS-STING DNA sensing pathway, including HSV-1 VP22 (16), UL41 (17), VP24 (34), UL24 (35), and UL36 (36), as well as viral proteins encoded by Kaposi sarcoma herpesvirus (12), human cytomegalovirus (37, 38), and murine gammaherpesvirus 68 (39). However, the strategies used by avian herpesviruses to hinder DNA sensing in host cells remain unclear. In the present study, we provided the first evidence that DEV US3 protein kinase could inhibit the DNA-sensing signaling and the production of IFN-I, thereby contributing to the immune evasion of DEV during infection in ducks.

DEV replicates quickly in many cell types and tissues, establishing latent and persistent infection in ducks. DEV infection upregulates the expression of multiple PRRs and ISGs in ducks, indicating the activation of innate immune responses to restrict DEV infection (2). Nevertheless, DEV exhibits a broad cell tropism in ducks and causes lifelong infection, and it is hard for the host immune system to repress it. Thus, DEV must have evolved efficient strategies to evade the host innate antiviral responses. Duck STING is vital for duck IFN-I induction and plays an important role in the host defense against DEV infection (40). Moreover, the DEV VP16 protein inhibits DNA sensing by interacting with duck IRF7 (22). However, it remains unclear whether other DEV viral proteins are involved in the antagonization of the DNA sensing pathway. This study identified DEV protein kinase US3 as an efficient inhibitor of the cGAS-STING pathway, expanding the family of viral proteins blocking the DNA-sensing signaling.

The US3 protein kinase encoded by alphaherpesviruses is a multifunctional protein essential for virus infection and replication (41). Several cellular proteins, such as histone deacetylase 1 (HDAC-1), HDAC-2, β-catenin, and the type II kinesin motor protein KIF3A, have been identified as HSV-1 US3 substrates (42–44). The viral proteins UL31, UL34, gB, and dUTPase were also shown to be phosphorylated by HSV-1 US3 (45–47). US3 plays a pivotal role in cell-to-cell spread and virion nuclear egress in DEV (48). However, its role in regulating host antiviral innate immunity remains to be elucidated. In this study, we sought to identify the cellular substrates of DEV US3 in the DNA sensing pathway and to investigate the role of DEV US3 in the evasion of host innate immunity. We found that DEV US3 interacts with the activation domain of IRF7 and phosphorylates IRF7, which blocked the dimerization and nuclear translocation of IRF7, leading to the inhibition of DNA sensing signaling.

IRF3 and IRF7 play crucial roles in IFN-β production, because all signals converge at these transcription activators. Phosphorylation regulates the nuclear localization and activation of IRF3/IRF7 during IFN-I production (7). In the DNA sensing signaling pathway, TBK1 phosphorylates STING to recruit IRF3/IRF7, allowing TBK1 to phosphorylate IRF3/IRF7. The phosphorylated IRFs undergo a conformational change to form dimers, which then enter the nuclei to initiate IFN-I transcription. The phosphorylation sites Ser386 and Ser396 are critical in IRF3 dimerization and activation (49), and the phosphorylation of IRF3 at Ser97 is associated with IRF3 nuclear import (50). Our results revealed that DEV US3 interacts with the constitutive and virus activation domain of IRF7; this domain is essential for IRF7 nuclear trafficking (51). The interaction and phosphorylation of the activation domain of IRF7 might induce atypical or hyperphosphorylated forms of IRF7, thereby inhibiting its activation. Several other posttranslational modifications, such as ubiquitination, methylation, and SUMOylation, are also critical for IRF3 activation. These different modifications might be related to each other and cooperate to affect IRF3 activation (52). In the future, it will be important to determine whether US3-mediated IRF7 phosphorylation can affect other types of posttranslational modifications.

In summary, our findings suggest a new role for DEV protein kinase US3; it inhibits IFN-β production by selectively targeting IRF7 in the DNA sensing pathway. By binding to the activation domain of IRF7, US3 phosphorylates IRF7, leading to the inhibition of IRF7 activation and IFN-β induction. Our findings reveal an important mechanism whereby DEV evades the immune system. These findings increase our understanding of the pathogenesis of DEV and have the potential to accelerate the development of more effective control strategies.

## MATERIALS AND METHODS

### Cells, viruses, and antibodies.

Primary DEFs were prepared from 10-day-old specific-pathogen-free duck embryos and cultured in Dulbecco’s modified Eagle medium (DMEM) (Life Technologies, Grand Island, NY) supplemented with 5% fetal bovine serum (FBS) (Sigma-Aldrich, St. Louis, MO). HEK293T (ATCC CRL-3216) cells were cultured in DMEM containing 10% FBS (Sigma-Aldrich, St. Louis, MO). DEV CV (GenBank no. JQ673560) and HVT FC126 (GenBank no. AF291866) strains were propagated in DEFs prior to use in this study. Commercially available antibodies, including mouse anti-Flag, rabbit anti-HA, mouse anti-c-Myc, rabbit anti-c-Myc, and mouse anti-actin (Sigma-Aldrich, St. Louis, MO, USA), were used. Rabbit anti-IRF7 and rabbit anti-US3 antibodies were prepared in our laboratory. ISD and poly(dA-dT) were purchased from InvivoGen (San Diego, CA, USA).

### Plasmid construction.

The DEV US3 gene was amplified from the genome of the DEV CV strain and cloned into the pCAGGS expression vector. Plasmids encoding duck cGAS (GenBank no. XM_021271479), STING (GenBank no. XM_027468120), TBK1 (GenBank no. KY963947), and IRF7 (GenBank no. MG707077) were constructed by cloning the synthesized sequence into pCAGGS with a Flag or HA tag fused to the 3′ end. The duck IFN-β promoter luciferase reporter IFN-β-luc was constructed by inserting the fragment of the duck IFN-β promoter from position −96 to +1 into the pGL3-basic vector.

### Quantitative real-time PCR.

Total RNA was extracted using RNAiso Plus reagent (TaKaRa, Otsu, Japan). Reverse transcription was performed using a ReverTra Ace qPCR RT kit (Toyobo, Osaka, Japan). The quantity of each cDNA was determined by qPCR using the Thunderbird SYBR qPCR mix (Lucigen, Madison, WI, USA) and analyzed with the LightCycler 480 system (Roche, Basel, Switzerland). Specific primers for IFN-β, IL-6, Mx, and OASL (see Table S1 in the supplemental material) were synthesized by Invitrogen (Shanghai, China), and the relative mRNA levels of these genes were normalized to the duck β-actin mRNA level in each sample. The fold differences between the treated samples and mock-treated samples were calculated. To determine DEV and HVT genome copy numbers, total DNA was extracted using the AxyPrep BodyFluid viral DNA/RNA miniprep kit (Corning Life Sciences, Shanghai, China) and tested with qPCR by measuring the copy numbers of the DEV UL30 and HVT UL30 genes as the viral genome targets and the duck glyceraldehyde-3-phosphate dehydrogenase (GAPDH) gene as a reference. qPCR was performed under the following cycling conditions: 95°C for 1 min for initial denaturation, followed by 40 cycles of 95°C for 15 s for denaturation, 60°C for 1 min, and collection of PCR product signals. All controls and treated samples were examined in triplicate in the same plate.

### Transfection and dual-luciferase reporter assays.

To determine duck IFN-β promoter activity, DEFs were cotransfected with the firefly luciferase reporter plasmid (IFN-β Luc) and *Renilla* luciferase reporter pRL-TK, which served as an internal control, with or without expression plasmids as indicated, using the TransIT-X2 dynamic delivery system (Mirus, Madison, WI, USA). At 36 h posttransfection, cells were lysed and samples were assayed for firefly and *Renilla* luciferase activity using the dual-luciferase reporter assay system (Promega, Madison, WI, USA). Relative luciferase activity was normalized to *Renilla* luciferase activity. The reporter assays were repeated at least three times.

### Coimmunoprecipitation and Western blot assays.

The expression plasmids harboring Flag, HA, or Myc tags were transfected into HEK293T or DEFs using the TransIT-X2 dynamic delivery system (Mirus, Madison, WI, USA). At 36 h posttransfection, cells were lysed in ice-cold Pierce IP buffer containing a protease inhibitor cocktail (Thermo Fisher Scientific, Waltham, MA, USA). The lysates were obtained by centrifugation and incubated with the indicated antibodies at 4°C overnight. Protein G Sepharose beads (Roche) were added, and samples were incubated for another 6 h. The beads were washed six times with phosphate-buffered saline and boiled in sodium dodecyl sulfate loading buffer before analysis by Western blotting with the indicated antibodies. For Western blotting, whole-cell lysates were obtained by lysing cells in NP-40 lysis buffer (Beyotime, Beijing, China). Protein concentrations were determined with a bicinchoninic acid protein assay kit (Thermo Fisher Scientific). The proteins were separated by electrophoresis on 12% SDS-polyacrylamide gels, transferred onto nitrocellulose membranes, and incubated with the indicated primary antibodies. IRDye 680RD goat anti-rabbit IgG and IRDye 800CW goat anti-mouse IgG (LI-COR, Lincoln, NE, USA) were used as the secondary antibodies. Images were acquired with the Odyssey infrared imaging system (LI-COR Biosciences, Lincoln, NE, USA).

### Confocal imaging.

DEFs were transfected with the plasmids and then treated with ISD to induce the nuclear translocation of IRF7. For confocal imaging, cells were first fixed with 4% paraformaldehyde for 30 min and permeabilized with 0.1% Triton X-100 for 15 min, which was followed by blocking with 5% bovine serum albumin for 1 h. Then, the cells were incubated with rabbit anti-IRF7, rabbit anti-HA, or mouse anti-Flag antibodies for 1 h. The cells were washed five times and incubated with Alexa 546–anti-rabbit immunoglobulin and Alexa 488–anti-mouse immunoglobulin secondary antibodies (Abcam, Cambridge, UK). Finally, nuclei were stained with 4′,6-diamidino-2-phenylindole (DAPI; Sigma-Aldrich). After being washed five times, the cells were examined using a confocal microscope system (Zeiss LSM880, Oberkochen, Germany).

### *In vitro* kinase and dephosphorylation assays.

To detect US3 kinase activity, the cells transfected with the US3 expression plasmid and the plasmid encoding IRF7 were lysed, and the supernatants were obtained by centrifugation. The supernatants were then loaded onto a phosphate gel for immunoblotting with anti-IRF7 antibodies. The phosphate gel was a 10% acrylamide gel containing 5 mM phosphate binding tag (Phos-tag; ApexBio Technology, USA) and a resolving gel with 10 mM Mn^2+^. The gel was washed three times every 10 min in transfer buffer containing 10 mM EDTA before transfer to increase the transfer efficiency. Phos-tag and Mn^2+^ cooperate to bind a phosphorylated protein; when the phosphorylation levels are increased, the migration velocity of the protein is lower. Therefore, nonphosphorylated and phosphorylated proteins can be separated in the gel. To confirm that DEV US3 mediates the phosphorylation of IRF7, cell lysates were subjected to a dephosphorylation assay using lambda protein phosphatase (PP) (New England Biolabs) (31). The samples with or without lambda PP treatment were then analyzed by SDS-PAGE and Western blot assays.

### Knockdown of US3 by RNA interference.

The following small interfering RNAs (siRNAs) were designed and used to downregulate DEV-US3: US3-siRNA-003, 5′-GGA AAC GUG UCA UAC CGA UTT-3′ (sense) and 5′-AUC GGU AUG ACA CGU UUC CTT-3′ (antisense); US3-siRNA-265, 5′-GCG GUU GUG AAU ACG CAA UTT-3′ (sense) and 5′-AUU GCG UAU UCA CAA CCG CTT-3′ (antisense); US3-siRNA-606, 5′-GCA CGG AAG AGG AAU AAU ATT-3′ (sense) and 5′-UAU UAU UCC UCU UCC GUG CTT-3′ (antisense); and US3-siRNA-958, 5′-GGU AAC UGC CGU AUU GUA ATT-3′ (sense) and 5′-UUA CAA UAC GGC AGU UAC CTT-3′ (antisense). All siRNAs were synthesized by GenePharma (Shanghai, China). The siRNA transfections were performed in DEFs using a TransIT-X2 dynamic delivery system (Mirus, Madison, WI, USA) according to the manufacturer’s instructions. Twelve hours after transfection, cells were harvested or infected with DEV for further analysis. The knockdown efficiency of US3 was verified by Western blotting.

### DEV titration.

DEV titers were estimated by the median tissue culture infective dose (TCID_50_). DEFs were seeded in 96-well plates at a density of 1 × 10^5^ cells per well. Cells were infected with serial 10-fold DEV dilutions and incubated for 5 days at 37°C in a 5% CO_2_ atmosphere. Cell pathological changes were observed under microscope and recorded. Viral titers were calculated according to the Reed-Muench method (53).

### Statistical analysis.

The data are presented as means and standard deviations (SD). Statistical significance between groups was determined by Student’s *t* test with GraphPad Prism 7.0 software (La Jolla, CA, USA). Statistical significance was set at a *P* value of <0.05.
