# Supplementary material for: Duck Enteritis Virus Protein Kinase US3 Inhibits DNA Sensing Signaling by Phosphorylating Interferon Regulatory Factor 7
Source: Microbiol Spectr. 2022 Oct 26;10(6):e02299-22. doi: 10.1128/spectrum.02299-22 (PMC9769898; doi:10.1128/spectrum.02299-22)
Supplement: Supplemental file 1 — Table S1. Download spectrum.02299-22-s0001.pdf, PDF file, 0.08 MB [file spectrum.02299-22-s0001.pdf]

TABLE S1 Sequences of primers used in the quantitative real-time PCR assay.

Primer name	Sequence (5' – 3')
IFN-β-F	TCTACAGAGCCTTGCCTGCAT
IFN-β-R	TGTCGGTGTCCAAAAGGATGT
IL-6-F	TTCGACGAGGAGAAATGCTT
IL-6-R	CCTTATCGTCGTTGCCAGAT
Mx-F	TGCTGTCCTTCATGACTTCG
Mx-R	GCTTTGCTGAGCCGATTAAC
OASL-F	TCTTCCTCAGCTGCTTCTCC
OASL-R	ACTTCGATGGACTCGCTGTT
β-actin-F	GATCACAGCCCTGGCACC
β-actin-R	CGGATTCATCATACTCCTGCTT
DEV-UL30-F	TTTCCTCCTCCTCGCTGAGTG
DEV-UL30-R	CCAGAAACATACTGTGAGAGT
HVT-UL30-F	TGCAGAACAAAGAGTATTCCGTA
HVT-UL30-R	CATCCCCTATCTTTACTAGCC
GAPDH-F	GTCAACGGATTTGGCCGTAT
GAPDH-R	CCACTTGGACTTTGCCAGAGA
